# Neutral Lipid Storage Diseases as Cellular Model to Study Lipid Droplet Function

**DOI:** 10.3390/cells8020187

**Published:** 2019-02-21

**Authors:** Sara Missaglia, Rosalind A. Coleman, Alvaro Mordente, Daniela Tavian

**Affiliations:** 1 Laboratory of Cellular Biochemistry and Molecular Biology, CRIBENS, Università Cattolica del Sacro Cuore, 20145 Milan, Italy; sara.missaglia@unicatt.it; 2 Psychology Department, Università Cattolica del Sacro Cuore, 20123 Milan, Italy; 3 Department of Nutrition, University of North Carolina, Chapel Hill, NC 27599, USA; rcoleman@unc.edu; 4 Dipartimento di Scienze di Laboratorio ed Infettivologiche, Fondazione Policlinico Universitario A. Gemelli IRCCS, 00168 Roma, Italy; Alvaro.Mordente@unicatt.it; 5 Facoltà di Scienze della Formazione, Università Cattolica del Sacro Cuore, 20123 Milano, Italy

**Keywords:** NLSD, *PNPLA2*, *ABHD5*, lipid metabolism, lipid droplet, myopathy, ichthyosis, cardiomyopathy, liver steatosis, Jordans’ anomaly, fibroblasts, induced pluripotent stem cells

## Abstract

Neutral lipid storage disease with myopathy (NLSDM) and with ichthyosis (NLSDI) are rare autosomal recessive disorders caused by mutations in the *PNPLA2* and in the *ABHD5/CGI58* genes, respectively. These genes encode the adipose triglyceride lipase (ATGL) and α-β hydrolase domain 5 (ABHD5) proteins, which play key roles in the function of lipid droplets (LDs). LDs, the main cellular storage sites of triacylglycerols and sterol esters, are highly dynamic organelles. Indeed, LDs are critical for both lipid metabolism and energy homeostasis. Partial or total *PNPLA2* or *ABHD5/CGI58* knockdown is characteristic of the cells of NLSD patients; thus, these cells are natural models with which one can unravel LD function. In this review we firstly summarize genetic and clinical data collected from NLSD patients, focusing particularly on muscle, skin, heart, and liver damage due to impaired LD function. Then, we discuss how NLSD cells were used to investigate and expand the current structural and functional knowledge of LDs.

## 1. Introduction

The human body stores excess amounts of biochemical energy in the form of triacylglycerols (TAGs). Together with cholesteryl esters (CEs), TAGs represent the vast majority of neutral lipids that are deposited in cytoplasmic inclusions known as lipid droplets (LDs) [1]. Adipose tissue is the main site for storage of TAGs, from which fatty acids (FAs) are released during periods of increased energy demand. In addition to adipocytes, all cell types can generate LDs in response to elevated FA levels, and subsequently utilize stored lipids when the conditions are reversed [2]. Indeed, LDs represent a fundamental component of intracellular lipid homeostasis, a universal feature of eukaryotic cells that can provide a rapidly mobilized lipid source for many important biochemical processes [3,4]. During starvation, FAs mobilized from LDs can be used for energy production via β-oxidation in the mitochondria or peroxisomes [5,6].

Excessive accumulation of neutral lipids in human cells results in common metabolic disorders, such as obesity and lipodystrophy, which are associated with dyslipidemia, insulin resistance, type 2 diabetes, hypertension, and coronary heart disease [7]. Moreover, LD accumulation in macrophages is a key feature of both early and late stages of atherosclerotic lesions [8]. LDs play also an important role in the inflammatory response, which may further link these sub-cellular structures to the development of both insulin resistance and atherosclerosis [9]. In addition to these multi-factorial diseases, some rare inherited disorders arising from defects in LD metabolism lead to a systemic increase in the size and number of these cytosolic inclusions. Neutral lipid storage diseases (NLSDs) are genetic disorders caused by mutations in adipose triglyceride lipase (ATGL/PNPLA2) or in α-β hydrolase domain 5 (CGI-58/ABHD5) [10,11]. NLSD patients are characterized by accumulation of TAG-rich cytosolic LDs in leucocytes, fibroblasts, keratinocytes, skeletal and cardiac myocytes, hepatocytes, and several other cell types, including some of central nervous and auditory systems.

Growing evidence indicates that LDs also play key roles in the cellular handling of other lipids and hydrophobic molecules, such as the storage of hydrophobic vitamins and signaling precursors that are not related to energy homeostasis [12,13]. In the human body, the major storage site of vitamin A (retinol) is in LDs within hepatic stellate cells, ensuring a constant supply of the vitamin during dietary insufficiency. Vitamin A is esterified with FAs and is stored in LDs as retinyl esters. Small amounts of vitamin A accumulate in LDs of extrahepatic stellate cells, as well as in hepatocytes and adipocytes [14]. Although adipocytes also contain vitamin E (tocopherol), mechanisms of storage, release, and function remain unclear [15]. The endocrine cells of gonads and adrenals accumulate steroid hormones and signaling FA, and eicosanoid precursors are found in LDs in activated mast cells [16,17]. Neutral lipids, stored in LDs, can also provide essential precursors used for membrane biogenesis and to support organelle or cell growth. Recent lipidomic analyses show that more than 100 different species of neutral lipids can be stored in the LD core, suggesting that significant intracellular exchanges occur to and from LDs [18].

LDs are also able to sequester endogenous and exogenous lipophilic compounds that can be toxic to the cells [19,20]. Moreover, LDs often function to ameliorate or mitigate endoplasmic reticulum (ER) stress, sequestering harmful lipids and misfolded ER proteins, as well as balancing lipid homeostasis in membranes [21,22]. The accumulation of LDs is associated with oxidative stress in cancer and non-alcoholic fatty liver disease [23,24]. Despite questions about this role, LD biogenesis can lower reactive oxygen species (ROS) levels in human cancer.

Other biological functions were recently linked to LDs, including protein maturation, storage, and degradation. This feature is observed in some viral proteins that must associate with the surface of LDs before correct maturation and incorporation into viral particles. The best studied example is represented by viral hepatitis C virus (HCV) proteins, but dengue virus and rotaviruses also exploit LDs for viral protein maturation [25,26,27]. In LDs, protein storage is well documented for histones in insects (*Drosophila*). In mammals (mice), recent studies showed that perilipin 5 (PLIN5) can be temporarily stored in LDs and then translocated to the nucleus in order to induce gene expression [28]. Another transcription factor, nuclear factor of activated T-cells 5 (NAFT5), can accumulate in LDs before being transferred to the nucleoplasm where it regulates the expression of osmoprotective genes in several lines of mammalian cells [29]. Finally, LDs might function as a specific platform for the turnover of highly hydrophobic proteins (ApoB, ApoB100) via proteasomal degradation or for the delivery of damaged proteins to the lysosome for autophagy [30].

LDs are generally considered to be cytoplasmic organelles. Although LDs are scarcely detected in cell nuclei of HeLa cells, fibroblasts, or differentiated adipocytes, many researchers readily observed LDs in the nuclei of human hepatocytes. Nuclear LDs are smaller than cytoplasmic ones, and present only a partial overlap in protein composition. Their physiological role remains to be clarified [31]. 

Several novel LD functions were revealed by recent studies. In this review, we summarize genetic and clinical data on NLSD patients, and we describe how cells of NLSD patients were extensively used to perform biochemical investigations in order to understand metabolic disturbances. At the same time, we focus the attention on NLSD cells as an excellent model for the study of further LD functions that are gradually emerging beyond energy homeostasis.

## 2. Neutral Lipid Storage Disorders

NLSD is a heterogeneous group of inherited disorders, comprising two autosomal recessive diseases: NLSD with myopathy (NLSDM) and NLSD with ichthyosis (NLSDI). The first NLSD patients were reported in 1953 by Jordans who described two brothers with progressive muscular dystrophy and “fat-containing vacuoles” in the leucocytes of peripheral blood [32]. These patients did not present with ichthyosis and, on the bases of clinical description, they were probably NLSDM patients. More than ten years later, Rozenszajn et al. reported two sisters with ichthyosis and many vacuoles in leucocytes, as well as in cells of myeloid origin [33]. These women would be now classified as NLSDI patients. In 1997, Igal et al. designated NLSD patients as representing each of the two phenotypic forms [34]. Since then, 55 NLSDM and 129 NLSDI patients were reported worldwide.

### 2.1. Neutral Lipid Storage Disorder with Myopathy

NLSDM (OMIM#610717) is a rare disorder of lipid metabolism caused by mutation in the patatin-like phospholipase domain containing 2 (*PNPLA2*) gene [30]. NLSDM patients are primarily affected by progressive myopathy, cardiomyopathy, hepatomegaly, diabetes, chronic pancreatitis, and short stature [35,36,37,38]. The clinical severity appears to be highly variable. Progressive skeletal muscle myopathy is always present, with both proximal and distal distribution. Asymmetric muscle involvement was reported in almost half of patients, with the right side mainly affected. Cardiac disfunction was observed in 40% of patients (22 of 55 subjects) with clinical manifestations ranging from minimal symptoms to severe conditions. Liver involvement was reported only in 20% of patients, mainly manifesting as hepatomegaly.

**Figure 1:**
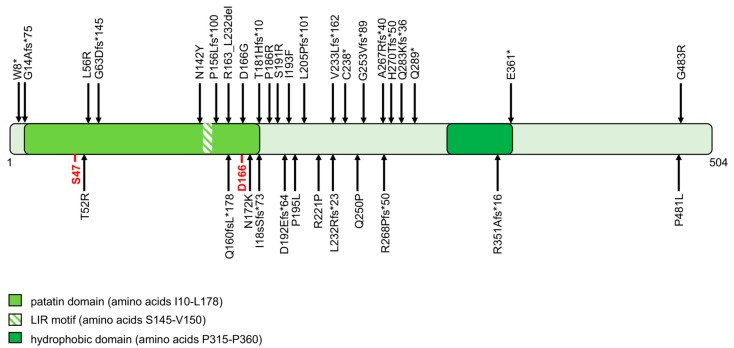
Structural domains of adipose triglyceride lipase (ATGL) protein and mutations identified in neutral lipid storage disease with myopathy (NLSDM) patients. The ATGL protein, consisting of 504 amino acids, comprises two functional regions: (i) the patatin domain, containing a catalytic site (S47 and D166, reported in red) and an LC3-interactinig region (LIR) motif; (ii) a hydrophobic region in the C terminal part, involved in lipid droplet (LD) binding. All ATGL protein mutations are reported in the scheme, based on recommendations for sequence variant description of Human Genome Variation Society (HGVS)-nomenclature website (http://varnomen.hgvs.org). Four *PNPLA2* mutations that cause no protein production (c.553_565delGTCCCCCTTCTCG; IVS6 + 1G > T; IVS6 + 2T > C; retrotransposon insertion) are not shown in the scheme.

From a genetic point of view, NLSDM was recently recognized and, even among experts, its clinical, genetic, and metabolic implications are yet to be completely elucidated [30,38]. *PNPLA2* gene mutations cause the onset of NLSDM. *PNPLA2* encodes for the protein adipose triglyceride lipase (ATGL). The human ATGL protein comprises 504 amino acids and contains a patatin domain located within the NH_2_-terminal region of the protein (Figure 1). The active site is characterized by a catalytic dyad (amino acid residues Ser47 and Asp166), within the patatin domain. The COOH-terminal region of ATGL contains a hydrophobic stretch (315–360 residues) required for binding to LDs. This enzyme catalyzes the first step in the hydrolysis of TAG, generating free FAs and diacylglycerol. Fifty-five patients were clinically and genetically characterized worldwide. The 39 different ATGL mutations reported differently affect protein function or production; 25 of the 39 (64%) result in truncated proteins (stop codon, frameshift, and splice site mutations), one is expected to abrogate protein expression, and the remaining 13 (33%) are missense mutations (Figure 1) [39,40,41,42,43,44,45,46,47,48,49,50,51,52]. 

**Figure 2:**
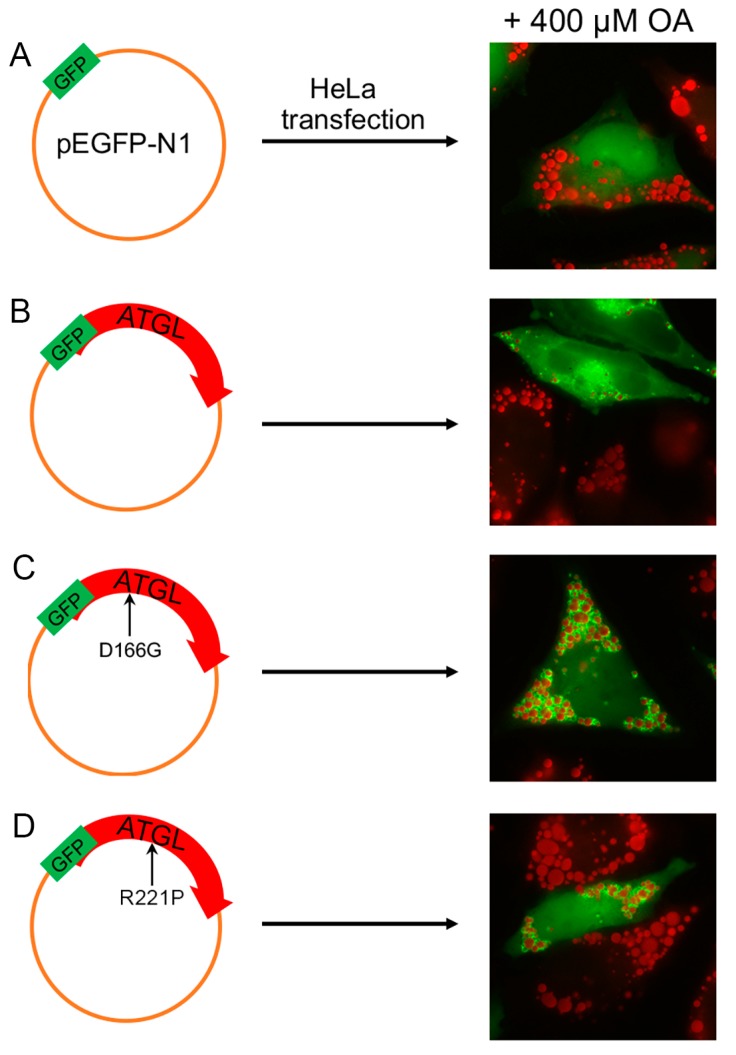
Qualitative and quantitative evaluation of LDs in HeLa cells transiently transfected with ATGL wild-type and mutant proteins. After incubation for 18 h with oleic acid (OA) (400 μM) complexed to bovine serum albumin (BSA) (6:1 molar ratio), HeLa cells were transiently transfected with either phosphor (p) enhanced GFP (EGFP; **A**), pATGL-EGFP (**B**), pATGL(D166G)-EGFP (**C**), or pATGL(R221P)-pEGFP (**D**). After 24 h, the cells were fixed and stained with Oil red O (ORO). Immunofluorescent imaging reveals that all ATGL proteins correctly localized to LDs. Quantification of LD number and size per cell was performed using the public-domain Java image-processing program WCIF ImageJ 1.35j (developed by W. Rasband; NIH, Bethesda, Maryland). Fluorescence of EGFP and ATGL-EGFP fusion proteins is shown in green. Magnification: 40×. These data were previously published in a different format [55].

All molecular data collected from NLSDM families suggest a marked genetic heterogeneity for this disease. At the moment, one cannot compare clinical data with a single genetic mutation, because most families present “private mutations” (Figure 1) and only a limited number of patients were reported. However, some functional studies based on the analysis of ATGL enzymatic activity were developed [53,54,55,56,57]. These assays were used to evaluate the pathogenic charge of *PNPLA2* missense mutations identified in NLSDM patients. In six of 13 (46%) missense variations, the ATGL mutated proteins were able to bind LDs, but the amino acid changes differently affected lipase activity (Figure 2). Reported findings provide evidence that the NLSDM patients who carry missense mutations manifest a mild disease phenotype, especially considering cardiac symptoms, with the exception of the patient in which a missense mutation disrupted the ATGL catalytic site [54]. Future molecular and functional analyses of *PNPLA2* missense mutations might be useful to explain variations in clinical expression of this syndrome.

### 2.2. Neutral Lipid Storage Disorder with Ichthyosis

**Figure 3:**
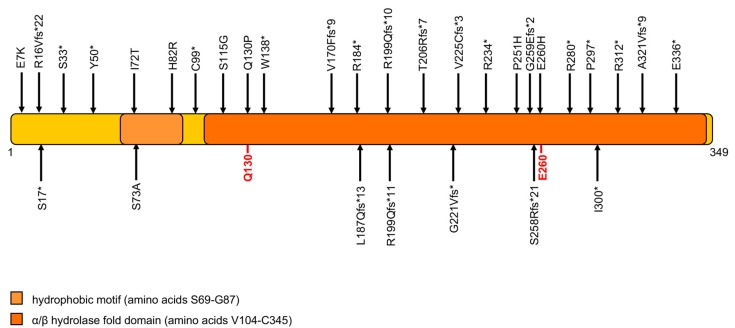
Domain organization of the α-β hydrolase domain 5 (ABHD5) protein, and variations associated with NLSD with ichthyosis (NLSDI). ABHD5 comprises 349 amino acids, characterized by an LD-binding site (hydrophobic domain) and an α/β hydrolase domain that includes two residues involved in ATGL and perilipin interaction (Q130 and E260). All ABHD5 mutations are shown based on recommendations for sequence variant description of HGVS-nomenclature website. Five variations (g.43728907_43732862del3955ins26, c.135 - 2A > G; c.506 - 3C > G; c.507-1G > A; IVS6 + 6A > T), that alter or abrogate RNA expression are not shown in the scheme.

NLSDI was genetically characterized in 2001 (OMIM#275630), when mutations of *ABHD5/CGI-58* gene were identified in patients characterized by a form of non-bullous congenital ichthyosiform erithroderma (NCIE) and the presence of intracellular LDs in most tissues [31]. The disease is historically known as Chanarin Dorfman syndrome (CDS) [58,59]. The clinical phenotype involves multiple organs and systems, including skeletal muscle, liver, eyes, ears, and the central nervous system [31,38,60]. Patients are sometimes born as collodion babies. While ichthyosis is always present, others clinical features may vary. Liver involvement is observed in greater than 80% of patients, ranging from hepatomegaly or liver steatosis to cirrhosis [31,61,62,63]. Sensorineural hearing loss is present in almost 30% of NLSDI patients. Myopathy usually begins in the 30s and muscle abnormalities can be detected in 40% of subjects [60,64]. Unlike NLSDM patients, NLSDI patients do not develop cardiomyopathy, probably because their cardiomyocytes have a limited but sufficient amount of energy. Although the ABHD5 protein promotes ATGL activation, ATGL is able to hydrolase TAG in the complete absence of ABHD5 protein, although with low efficiency [65]. ABHD5 is a 349-amino-acid-long protein characterized by a lipid-binding motif and a hydrolase domain containing Q130 and E260 amino acids, which are essential residues for ATGL interaction (Figure 3).

To date, 129 NLSDI patients were reported. For 85 of these patients, clinical diagnosis was confirmed by ABHD5 mutation analysis. Furthermore, 80% of ABHD5 mutations (nonsense, frameshift and splice site mutations) determine the production of truncated proteins, most of which are missing a large portion of the protein and the E260 amino acid residue, which allows ATGL interaction [31,60,62]. Missense mutations were identified in 20% of NLSDI patients. There are no hotspot mutations in the *ABHD5* gene, but the c.594insC variation was often detected (in 24 out of the 85 patients with molecular diagnosis), especially in Turkish patients [64,66]. Ninety NLSDI families were described worldwide; most are from Turkey, where a high incidence of consanguineous marriage occurs. The c.594insC mutation leads to a frameshift that produces a premature termination of translation and the formation of a truncated protein lacking 150 amino acids in the C-terminal region (p.R199Qfs*10). Although this mutation is present in 28% of NLSDI patients, no molecular investigation was performed to evaluate whether this truncated protein is expressed in patients’ cells, whether it is still able to localize to LDs, or whether it maintains residual protein activity.

### 2.3. ATGL and ABHD5 Proteins in LD

**Figure 4:**
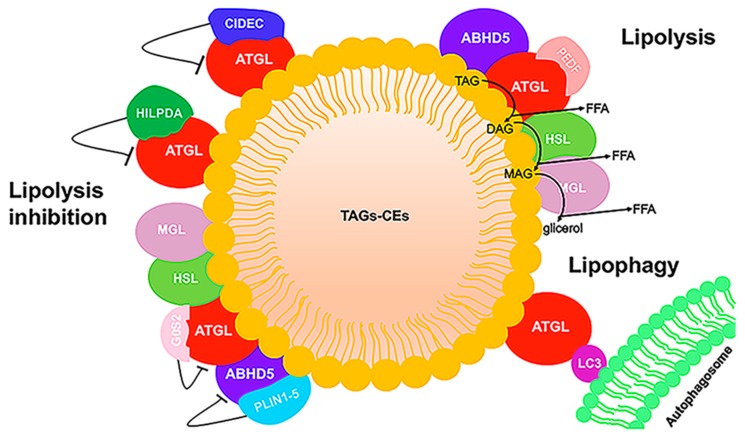
LD schematic representation. LDs have a core composed of triacylglycerol (TAG) and cholesteryl ester (CE), surrounded by a phospholipid monolayer. Some proteins binding to the phospholipid monolayer are involved in neutral lipid metabolism. In the stimulated condition, ABHD5 binds to and activates ATGL, and TAG is hydrolyzed sequentially by ATGL, hormone-sensitive lipase (HSL), and monoacyl-glycerol lipase (MGL) to generate fatty acid (FA) and glycerol. In hepatic and muscle cells, pigment epithelium-derived factor (PEDF) stimulates lipolysis by binding ATGL. In the unstimulated state, lipolysis is inhibited by the association of ATGL with G0/G1 switch gene 2 (G0S2), hypoxia-inducible LD-associated protein (HILPDA), and cell death activator CIDE-3 (CIDEC) and by the interaction between ABHD5 and perilipin (PLIN) proteins. In addition, ATGL can interact with LC3, an autophagic protein. Direct ATGL–LC3 binding can facilitate correct ATGL localization on the LD surface.

Lipid droplets were long considered to be inert fat depots. During the last decade, however, studies underlined the pleiotropic function of LDs, ranging from energy storage to regulation of lipid trafficking, response to oxidative stress, endoplasmic reticulum stress (ER), protein management, viral replication, activation of autophagy, mitochondrial dysfunction, inflammation, and cell death [67]. LDs present a unique structure among organelles in that they have a neutral lipid-central core and a phospholipid monolayer with many associated peripheral and integral proteins [68,69]. Emerging evidence suggests that, in many tissues, the droplet pools show different protein composition and functions [68,70]. In this review, we focus on the ATGL and ABHD5 proteins and their interaction with other LD proteins involved in lipolysis, a highly regulated process causing the release of FAs from TAGs. Mechanisms that control LD breakdown are critical for energy production mediated by FA and FA trafficking. ATGL catalyzes the rate-limiting step in TAG lipolysis that is then followed by the action of hormone-sensitive lipase (HSL) and monoacyl-glycerol lipase (MGL). In adipocytes, lipolysis is hormonally regulated by catecholamines, and releases FAs that are sent to non-adipose tissues during fasting for mitochondrial energy production. In all non-adipose cells (fibroblasts, hepatocytes, macrophages, myocytes), mobilization of stored TAG is regulated differently and is involved in multiple processes, including FA oxidation, lipid mediator synthesis, cell growth, membrane synthesis, and ER homeostasis [71]. ABHD5 co-activation of ATGL-mediated TAG hydrolysis is well described in multiple tissues. In adipocytes, the low basal level of lipolysis is due to the constitutive action of ATGL. Indeed, ATGL is associated with the LD surface through its hydrophobic domain and it is bound to the G0/G1 switch gene 2 (G0/S2) protein which partially inhibits its hydrolase activity (Figure 4) [72].

In unstimulated conditions, perilipin 1 (PLIN1), the first identified LD-associated protein, sequesters ABHD5 together with small amounts of HSL on the LD surface, thereby reducing lipolysis. Hormone stimulation induces protein kinase A (PKA) activation which in turn causes the hyper-phosphorylation of PLIN1. This results in the release of ABHD5 from PLIN1 and the subsequent interaction between ATGL and ABHD5, which increases the catalytic activity of ATGL. Moreover, PKA also phosphorylates HSL, which translocates to the LD surface where it hydrolyzes diacylglycerol (DAG) [73]. The perilipin proteins (PLIN1–5) are the major LD-associated proteins and are considered to be markers for LDs [74,75]. They form a barrier that prevents the access of ATGL to LD TAG. While PLIN1 is prevalently expressed in adipocytes, PLIN2 (also known as ADFP or ADRP) and PLIN3 are ubiquitously expressed. Both PLIN2 and PLIN3 lack the C-terminal part of PLIN1 required for binding with ABHD5. It was demonstrated that, upon lipolytic stimulation, PLIN2 and PLIN3 undergo proteasome degradation. This depletion facilitates the association of ATGL with LDs and with ABHD5 [76]. PLIN4 is primarily expressed in adipose tissue, but also in heart and skeletal muscle. The role of the PLIN4 protein remains unclarified. In skeletal muscle and in the heart, PLIN5 responds to PKA stimulation, and its phosphorylation causes the displacement of ABHD5 from PLIN5, allowing ABHD5 to bind to ATGL and increase the rate of TAG hydrolysis [77]. Thus, PLIN5 regulates ATGL activity in oxidative tissues similar to the role of PLIN1 in adipose tissue.

ATGL’s enzymatic activity is activated by ABHD5 and inhibited by the G0S2 protein. G0S2 inhibits ATGL in a dose-dependent non-competitive manner in the presence of its coactivator ABHD5. The 254 N-terminal residues of ATGL are required for TAG hydrolase activity and for interaction with the co-activator ABHD5 and the inhibitor G0S2 [78].

Other protein interaction partners of ATGL were reported, but their mechanisms of interaction remain to be elucidated. Proteomics and functional analyses identified a small protein encoded by hypoxia-inducible gene 2 (HIG2), also known as hypoxia-inducible LD-associated protein (HILPDA), which seems to act as a new inhibitor of ATGL, directly binding to the patatin domain [79]. This protein strongly inhibits ATGL-mediated lipolysis when cells are in hypoxic conditions. Another ATGL regulator is pigment epithelium-derived factor (PEDF), also known as serpin family F member 1 (SERPINF1). PEDF stimulates TAG hydrolase activity in liver and muscle lysates in vitro and increases the release of FAs from adipocytes in an ATGL-dependent manner [80]. The role of PEDF in lipid metabolism remains largely unknown, but some clinical studies suggest a positive correlation between serum PEDF levels and several major metabolic disorders. Cell death activator CIDE-3 (CIDEC) is also considered to be an LD-associated protein involved in ATGL regulation [76]. It promotes LD enlargement by preventing access of ATGL to TAG stored in LDs. Indeed, the C-terminus of CIDEC binds ATGL and is also responsible for interaction with PLIN1, supporting its co-localization on the LD surface [76]. 

LDs are broken down through two different mechanisms: lipolysis and autophagy/lipophagy. These two processes are partially interconnected, and ATGL is a player common to each. For example, emerging studies show that LC3, a well-known marker of the autophagosome, interacts with ATGL at the LD surface. Indeed, ATGL and HSL each exhibit multiple LC3-interacting region (LIR) motifs. Mutating these LIRs reduces basal ATGL localization to LDs in response to serum starvation, thereby preventing the translocation of ATGL to the LD surface. Although these studies suggest that lipophagy and LC3-regulated ATGL activity are complementary in total lipolysis, the findings do not fully explain why ATGL needs LC3 for LD localization [81]. 

## 3. LDs in NLSD Patient Tissues

Under normal conditions, all of the cell types that were examined so far have the ability to generate LDs in response to elevated FA levels and, if no metabolic defects are present, to disperse these lipid structures, when the conditions are reversed. In NLSD cells, a deficit in the degradation of cytoplasmic TAG prevents FA mobilization. The dysregulation of TAG breakdown produces an abnormal accumulation of neutral lipids in all tissues, including skeletal muscle, heart, liver, and skin, and determines the formation of LDs that are more numerous and larger than those observed in control cells.

### 3.1. Jordans’ Anomaly

**Figure 5:**
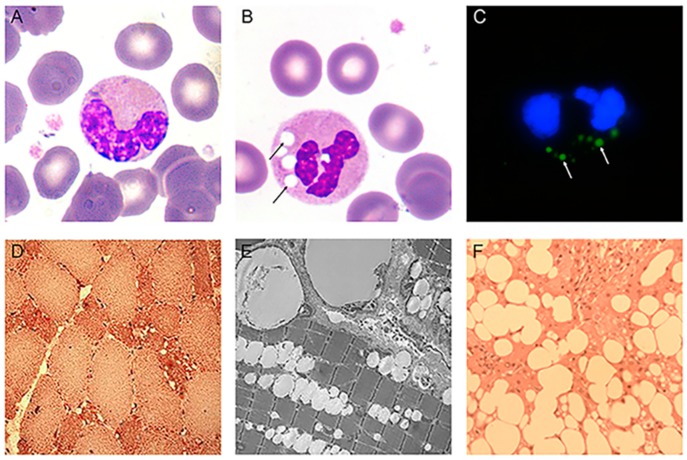
Evaluation of abnormal LDs in specific tissues. Microphotographs of Jordans’ anomaly, indicated by arrows, in NLSD patient buffy coats stained with May-Grunwald-Giemsa (MGG) (**B**) compared to control cells (**A**) (magnification 100×). (**C**) Immunofluorescent imagine of NLSD granulocytes stained with Bodipy493/503 (Jordans’ anomaly) and DAPI (nuclei) (magnification 100×). (**D**) Histochemical characterization of NLSDM quadriceps muscle biopsy, stained with Oil red O (ORO). Abnormal storage of neutral lipids is primarily detected in hypotrophic type I fibers. Image courtesy of Prof. Marina Mora (Fondazione IRCCS Istituto Neurologico “Carlo Besta”, Italy). (**E**) Electron microphotographs of human quadriceps muscle from a subject with NLSDM. Large LDs are located near to mitochondria and the subsarcolemmal region. Image courtesy of Prof. Marina Mora (Fondazione IRCCS Istituto Neurologico “Carlo Besta”, Italy). (**F**) Diffuse macrovesicular steatosis (ORO staining) in a liver biopsy from a subject with NLSDI. Image courtesy of Prof. Eugenia Valadares (Department of Propedêutica Complementar, Universidade Federal de Minas Gerais, Belo Horizonte, Brazil).

Since the first observation of unusual vacuoles, called Jordans’ anomalies, in the cytoplasm of granulocytes of NLSD patients [32], this feature became the most common laboratory finding for the clinical diagnosis of NLSD [35,38,64,66]. May-Grunwald-Giemsa (MGG) stain was originally used to detect Jordans’ anomalies, and it remains the fastest and most reliable method to detect these vacuoles. In this simple method, buffy coats obtained from fresh ethylenediaminetetraacetic acid (EDTA)-treated peripheral blood samples of patients are smeared onto slide glasses and treated with MGG reagents. After staining, round and bright vacuoles appear in the cytoplasm (Figure 5B). In 1966, cytochemical analysis showed that the inclusions observed in NLSD granulocytes were positive for Sudan III, Neutral Red, and Rhodamine B staining [33]. This result demonstrated that the vacuoles contained neutral lipids. The TAG content of NLSD granulocytes was two to three times greater than in control cells, further supporting the cytochemical studies [58].

The first investigation of Jordans’ anomalies in blood cells revealed that the vacuoles were also present in promyelocytes and that their number increased during cell lifespan [33]. This evidence suggested that the granulocytes begin to accumulate neutral lipids as they mature in the bone marrow.

Most NLSD patients show lipid-containing vacuoles in 80–100% of their white blood cells (182 patients) [38,60,82,83]. In two patients, only a small percentage of granulocytes with cytoplasmic inclusions was detected. The first one had 20–30% of blood cells with Jordans’ anomalies and was diagnosed with NLSDI based on clinical findings of congenital ichthyosis, hepatomegaly, and increased gamma glutamyl transferase (GGT) and amino transaminase. Complete genetic analysis of ABHD5 and its promotor region did not reveal any pathogenic variation [60,84], suggesting a possible genetic heterogeneity in NLSDI. The second patient (NLSDM) had Jordans’ anomalies in only 3–5% of granulocytes [57]. Molecular analysis revealed two *PNPLA2* mutations in heterozygous status: c.497A > G (p.D166G) and c.1442C > T (p.P481L). The first variation led to a complete loss of lipase activity, while the second was associated with a partial decrease in ATGL function. The p.P481L ATGL protein that retained lipase activity may have prevented TAG storage in white cells. 

Although MGG staining is the conventional procedure to detect vacuoles in NLSD granulocytes, the use of lipophilic dyes such as Nile Red, Oil red O, or Bodipy493/503 is preferred for a quantitative analysis of Jordans’ anomalies [85] (Figure 5C). These procedures also prevent the generation of staining artefacts due to MGG-negative spots caused by inclusions that do not contain neutral lipids. 

Jordans’ anomalies in NLSDM granulocytes can be analyzed with an automated hematology analyzer [86]. This instrument shows an increase in small lipid-containing particles released from granulocytes of ATGL-deficient patients compared to control. Although the efficacy of this method remains to be further tested, the authors stressed that this automated analysis could represent an effective clinical tool to provide an early diagnosis of ATGL deficiency. 

### 3.2. Muscle

TAG is the major neutral lipid stored in skeletal muscle. Most muscle LDs are located in subsarcolemmal region or between the myofibrils, usually near mitochondria. The organization of skeletal muscle fibers limits organelle mobility, including that of LDs; nevertheless, the high metabolic rate of skeletal muscle requires a certain degree of mobility to ensure the turnover of LDs and their interaction with other organelles, particularly with mitochondria. Skeletal muscle is subjected to high energetic demand and requires a large supply of TAGs for ATP production. After hydrolysis, FAs are transported to mitochondria where they undergo oxidative metabolism. A rapid transfer of energy sources is facilitated by a direct interaction between LDs and mitochondria, which avoids a high concentration of cytotoxic FAs in the cytosol [87]. In skeletal muscle, LDs and mitochondria have a physical relationship and are arranged alternately in rows. Several proteins were described at the LD–mitochondria contact site, including PLIN5, which is reported to induce recruitment of LDs and mitochondria toward each other [88]. LDs in skeletal muscle serve as energy depots and energy provision during exercise [89]. Type I (oxidative) muscle fibers contain the largest number and size of LDs (0.3–1.5 µm in diameter) [87]. ATGL and HSL together account for at least 98% of TAG hydrolysis, suggesting a potential but limited contribution of additional lipases in skeletal muscle. HSL has a preference for DAG hydrolysis, but it is also able to hydrolyze TAG. Recent studies suggest that autophagy could also contribute to lipolysis in muscle. The coat proteins PLIN2 and PLIN3 undergo chaperone-mediated autophagy before lipolysis. Indeed, the degradation of PLIN2 and PLIN3 allows ATGL to localize on the LDs. While it is known that autophagy is an important process during starvation, further research is needed to investigate the importance of autophagy for skeletal muscle lipolysis during exercise. Of note, FAs generated by lipolysis not only serve as an energy substrate but are also signaling molecules. In skeletal muscle, ATGL overexpression activates peroxisome proliferator-activated receptor δ (PPARδ) to increase peroxisome proliferator-activated receptor gamma coactivator 1-alpha (PGC1α) expression, mitochondrial capacity, and increased gene activation [87].

The main clinical feature of NLSDM is skeletal muscle myopathy, which is present in 100% of patients and is accompanied by muscular atrophy in advanced cases. Muscle weakness usually represents the first diagnostic symptom in early adult life [38]. The most obvious explanation of the muscle weakness is that the defective ATGL activity impairs lipolytic breakdown of muscular TAG stores, which alters energy production. In skeletal muscle biopsies of NLSDM patients, histological and electron microscopy findings show a markedly increased number of LDs in the muscle fibers, particularly in type I fibers (Figure 5D,E). This is probably due to the absence of ATGL activity, which is primarily detected in type I muscle fibers. Moreover, in NLSDM, these fibers are often small with loss of myofibrils. Electron microscopy reveals an impressive increase of LDs which are mostly present between myofibrils which can appear dislocated. Initially, it was hypothesized that large lipid droplets between myofibrils might mechanically impair the muscle filament contraction and cause progressive atrophy of muscle fibers. However, the massive lipid storage in muscle fibers does not interfere with contractile function or muscle energetics in some NLSD patients [38,45,48]. This observation raised some doubts on the mechanical role of storage material in causing muscle weakness. Additional potential causes for the myopathy are lipotoxicity and altered lipophagy, but the mechanisms that underlie muscle damage in NLSD patients remain mainly unexplored. 

Myopathy is reported in almost 40% of NLSDI patients and is associated with milder muscular damage than in NLSDM. This difference might be due to fact that, in NLSDM patients, ATGL function is directly impaired, while, in NLSDI, the coactivator of ATGL, ABHD5, is defective. However, ATGL is able to hydrolase TAG in the complete absence of the ABHD5 protein, although with lower efficiency [65]. Despite the different degree of clinical severity, muscle tissue histology in both NLSDM and NLSDI patients reveals the same vacuolization of fibers, without any increase in connective or adipose tissue [38]. Moreover, mitochondrial alteration or localization was not reported in these patients [51].

### 3.3. Heart

The heart is continuously contracting and requires a continuous supply of energy substrates. FAs contribute 60–70% of cardiac energy supply, with glucose covering the rest. Most of the FAs entering cardiomyocytes are initially esterified into TAG and stored in LDs before being oxidized. The size of LDs in cardiomyocytes is similar to that of LDs in skeletal myocytes. The enzymatic pathways regulating lipolysis of cardiac TAG are similar to those in adipocytes and skeletal myocytes. Unfortunately, LD metabolism in human cardiomyocytes is largely unexplored. The explanted heart of an NLSDM patient revealed numerous vacuoles positive for Oil red O in cardiomyocyte cytoplasm, and positive vacuoles were also found in the cytoplasm of endothelial cells, smooth muscle cells of the coronary arteries, and foam cells in the intima. The TAG content in the left ventricle was markedly increased as compared with that of subjects without heart disease [41]. The analyses of other explanted and autopsied hearts from NLSDM patients revealed defective intracellular hydrolysis of TAG which results in cardiomyocyte steatosis and congestive heart failure, as well as coronary atherosclerosis with TAG-deposit smooth muscle cells defined as TAG deposit cardiomyovasculopathy (TGCV) [37,41]. NLSDM subjects with cardiac involvement could present as adult-onset progressive heart failure, mimicking dilated or hypertrophic cardiomyopathy, and sometimes vasospastic angina pectoris. Cardiac damage was reported in almost 40% of NLSDM patients, and clinical variability is high. Indeed, cardiomyopathy was lethal in some patients or necessitated cardiac transplantation [90], but some NLSDM patients have less severe heart damage [44]. The different severity of cardiomyopathy may be due to different ATGL mutations that cause total or partial loss of lipase activity. Mutations that completely abrogate lipase function should cause a more severe phenotype than mutations that partially impair enzymatic activity, since long-chain FA (LCFA) is a major energy source for the heart and ATGL is the primary intracellular enzyme that releases LCFA from TAG [52,55,56].

Unlike NLSDM patients, NLSDI patients do not develop cardiomyopathy; their cardiomyocytes derive a smaller but sufficient amount of energy from basal ATGL lipase activity [55,91].

### 3.4. Liver

The liver is a critical hub for lipid metabolism. In both hepatocytes and other liver cells, neutral lipids are stored in cytoplasmic LDs. In the liver, hepatocytes are the major site of FA turnover, accounting for more than 90% of liver cells. Different conditions result in abnormal lipid accumulation in the liver. Imbalance in the processes that maintain normal homeostasis can lead to non-physiological accumulation of TAG in hepatocytes. An elevated and continuous flux of FAs from adipose tissue to the liver is the main cause for the development of non-alcoholic fatty liver disease (NAFLD) which is a huge public health problem [92]. However, decreased FA oxidation also causes an excess of TAG storage in LDs. Indeed, in NLSDI, where ABHD5 mutations cause the onset of the disease, liver is the most frequently damaged organ with hepatomegaly, steatosis, and sometimes cirrhosis. More than 80% of NLSDI patients have fatty livers. In control liver, ATGL is expressed at low levels in hepatocytes, hepatic stellate cells, and Kupffer cells, whereas ABHD5 messenger RNA (mRNA) is highly expressed. This suggests that ABHD5 might activate additional unidentified lipases in addition to ATGL or might have additional functions in liver [91]. Absence of the additional function could explain the higher risk of hepatic steatosis with defective ABHD5 than with defective ATGL. In NLSDI patients, liver biopsies reveal intra-hepatocyte lipid vacuoles that are much larger (several microns in diameter) than those found in control liver (0.5–2 µm) (Figure 5E). These LDs are both microvesicular (accumulation of small LDs in hepatocytes with preserved cellular architecture) and macrovesicular (larger droplets that displace the nucleus) [60,61,62,63]. Sometimes, both fibrosis and cirrhosis can be detected [38]. The factors determining the progression from steatosis to fibrosis and cirrhosis are yet to be elucidated. It is likely that fibrosis is induced by the activated hepatic stellate cells (HSCs) producing large amounts of extracellular matrix. This process depends on unbalanced lipid metabolism. In contrast, although almost 20% of NLSDM patients have mild hepatic steatosis and liver enlargement by ultrasound; histological examinations are yet to be performed on liver biopsies from these patients [37,38,56].

### 3.5. Skin

**Figure 6:**
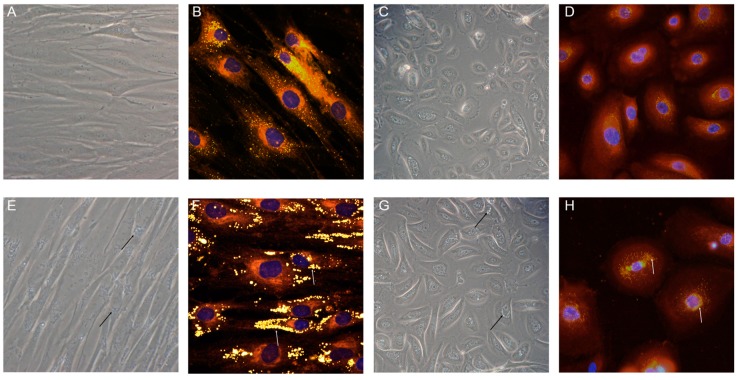
LD images obtained from NLSDI skin cells. Phase-contrast images of cultured fibroblasts and keratinocytes from control (**A**,**C**) and affected subjects (**E**,**G**); fluorescent microscopy images with Nile Red and DAPI staining of fibroblasts and keratinocytes from normal (**B**,**D**) and NLSDI (**F**,**H**) subjects. LDs are indicated by arrows. Phase-contrast images: magnification 20×; fluorescent microscopy images: magnification 40×.

Primary cultures of fibroblasts from NLSD patients represent an invaluable resource for investigating the molecular bases of pathology. Moreover, human NLSDM fibroblasts cell lines were reprogrammed in iPSC6 and could be a powerful means to establish translational platforms for disease modeling, drug discovery, and pre-clinical testing (for a detailed description, see Section 4). In NLSD fibroblasts and keratinocytes, the biochemical pathway concerning TAG hydrolysis via ABHD5–ATGL interaction is largely compromised. Indeed, a systemic increase in the size and number of LDs can be easily detected by phase-contrast microcopy or by neutral lipid staining (Figure 6). 

Although both NLSDI and NLSDM skin cells contain a high number of large-diameter cytoplasmic LDs, only patients with ABHD5 mutations exhibit a non-bullous congenital ichthyosiform erithroderma (NCIE) and are sometimes born as collodion babies [38,60]. In general, erythrodermic ichthyosis with fine, white, translucent, and semi-adherent scales is observed on the entire body of NLSDI subjects, particularly on the trunk, flexures, scalp, and face [58,60,64,66]. The face and limbs can present as gray-brown, polygonal, and adherent scales [64,93], and the skin of some patients shows erythematous plaques alternating with areas of uninvolved skin [94,95]. Rarely, ichthyosis may be accompanied by pruritus [96], and hyperkeratosis can be detected on the palms and soles [97].

Since the beginning of the research on myopathies associated with the accumulation of neutral lipids, the exclusive association of ichthyosis with NLSDI and not with NLSDM was recognized as a significant clinical difference between the two pathologies. Geneticists hypothesized that two genes, coding for proteins of the same enzymatic pathway, might be involved in the pathogenesis of the NLSD. Indeed, in the first decade of the 2000s, ATGL and ABHD5 were defined as the major contributors to the first step of TAG hydrolysis; at the same time, however, the lack of a skin defect in NLSDM patients suggested that ABHD5 had a function that was unrelated to ATGL activation. Two independent groups demonstrated that ABHD5 activates PNPLA1 (in addition to PNPLA2), which catalyzes the final step of ὠ-*O*-acylceramide production in human keratinocytes. ABHD5 recruits PNPLA1 to its putative TAG substrate localized on the LDs. The reported data highlight the molecular mechanism via which ABHD5 mutations cause the onset of ichthyosis in NLSDI, since ὠ-*O*-acylceramide is an essential lipid for skin permeability barrier formation [98,99]. These recent findings agree with immunoelectron microscopy observations of skin samples in which the ABHD5 protein was restricted to the granular layers (LGs) of keratinocytes in the upper spinous and granular layers of the epidermis in control subjects but not in NLSDI patients [100,101]. LGs are essential for skin barrier formation during keratinocyte differentiation because they transport lipids and other molecules. Moreover, Uchida et al. previously demonstrated a deficiency of ὠ-*O*-acylceramide in keratinocytes from NLSDI patients, and speculated that ABHD5 mutations could be responsible for the diminished synthesis of this essential skin barrier lipid [102]. 

## 4. NLSD Cellular Models

At present, no specific therapy exists for NLSD, and patients may develop serious and irreversible liver, muscle, and cardiac injury. The cells obtained from NLSD patients offer an opportunity to establish translational platforms for drug discovery and pre-clinical testing. Indeed, since the first investigations, NLSD fibroblasts represented a powerful tool to clarify molecular errors associated with abnormal storage of neutral lipid. Moreover, the generation of NLSDM iPSCs represents a powerful opportunity to derive specific cells (myocytes, cardiomyocytes, hepatocytes) in which the NLSD clinical phenotypes can be investigated.

### 4.1. Historical Experiments Performed on Primary Dermal NLSD Fibroblasts

Because the genetic defects associated with NLSDs were identified in 2001 [31] and 2007 [30], the first cellular studies aimed to clarify the metabolic defect that caused abnormal storage of TAGs. Preliminary observations showed that lipid vacuoles in NLSDI fibroblasts persisted when these cells were cultured with different media (lipid-free, conditioned from normal cells) [58,103] and that incubation with radiolabeled oleate showed abnormal storage of labeled lipids in the TAG fraction [58], as well as in free FAs, and monoacyl- and diacylglycerol fractions [104]. These data were further supported by a study of lipid metabolism in three NLSDI fibroblast cell lines [105]. In these cells, cultured under the same conditions as normal fibroblasts, TAG content was 10–20-fold higher than that in the control cells, and the abnormal accumulation did not decrease in the presence of lipoprotein-depleted media or solvent-delipidated serum. Analysis of control and NLSDI fibroblasts revealed an identical FA composition, apart from a defect on the catabolism of specific FAs. Moreover, evaluation of acid lipase activity in cells from normal subjects and those with NLSDI or Wolman’s disease (impaired lysosomal acid lipase activity with TAGs and CEs) showed no significant deficiency of acid lipase in NLSDI fibroblasts. Finally, normal oxidation levels, determined after incubation with [^14^C]-palmitate for one hour, indicated no defect in FA uptake, cytoplasmic transport, transmitochondrial transport, or activation. In another study, a pulse-chase experiment demonstrated that TAGs were not degraded at the end of chase period in NLSDM fibroblasts [106]. This was the first suggestion that the accumulation of neutral lipid in NLSD cells was due to a defect in TAG hydrolysis. 

At this time, cellular studies were carried out to determine whether TAG and CE were degraded via the same pathway [107]. Two NLSDM and three lines of control fibroblasts were incubated with [^3^H]-oleic acid for 12 hours to obtain TAG and CE from endogenous biosynthesis, and then with high-density lipoprotein (HDL)-[^3^H]-triolein and cholesteryl [^14^C]-oleate for 24 hours, to produce neutral lipids from uptake of exogenous HDL. At the end of pulse phase, the amounts of radiolabeled TAG and CE were analyzed, and time of degradation was measured. During chase periods, labeled TAG decreased slowly in NLSDM fibroblasts, but the degradation of radiolabeled CE in normal and NLSDM fibroblasts was similar. These results suggested that TAG and CE were degraded via two different pathways and that the degradation pathway of endogenous and HDL-TAGs was defective in NLSDM. Moreover, a comparative study using fibroblasts from subjects with Wolman’s disease or NLSDM demonstrated that the defect in HDL-TAG degradation did not correlate with lysosome in NLSDM cells, and that endogenously synthesized and HDL-TAGs were hydrolyzed within the same enzymatic pool [108]. 

In 1996 and 1998, additional experiments clarified the impact of defective TAG metabolism on phospholipid synthesis in NLSDI cells [109,110]. Compared to control cells, NLSDI fibroblasts showed not only an increase in TAG synthesis, but also severe modifications in synthesis and degradation of major phospholipids. In particular, an elevated synthesis of phosphatidylcholine, phosphatidylserine, phosphatidylinositol, and sphingomyelin was observed, but phosphatidylethanolamine synthesis was reduced. These data suggested that NLSDI cells were impaired in regulatory mechanisms of phospholipids turnover.

Taken as a whole, these studies identified a defect in TAG turnover as the major metabolic defect in NLSD onset, and showed that abnormal TAG storage involved neither lysosome nor acid lipase but some other novel enzymatic pathway.

### 4.2. Studies of Cultured Fibroblasts

Because biochemical studies performed on NLSD cells identified a TAG turnover defect, the genetic investigation focused on genes involved in TAG hydrolysis. Interspecies homology comparisons and linkage and haplotype analysis led to identification of ABHD5 mutations as the cause of NLSDI [31], and, in 2004, a novel TAG lipase, named ATGL, was independently identified by three different groups [111,112,113]. This enzyme was highly expressed in adipocytes but also, at lower levels, in other tissues, including skeletal and cardiac muscle [109]. Moreover, ATGL was downregulated in animal models of obesity and diabetes [112]. Taken together, these data suggested that ATGL could play a key role in lipid metabolism, particularly in TAG breakdown. In 2007, the identification of ATGL mutations in three NLSD patients without ichthyosis confirmed the involvement of ATGL in NLSDM [30].

After identification of genetic alterations implicated in NLSD, functional characterization of NLSD-causing mutations [55,56,57] and investigation of the possible phenotype–genotype correlation [37] were carried out. Moreover, an evaluation of strategies to overcome or correct the impairment in TAG metabolism was performed [35,42,55]. Fibroblasts obtained from NLSD patients were used as cellular models, because, in the absence of ATGL or CGI58, these cells accumulate a large number of LDs that can be readily detected and quantified [55,60]. Two beta-adrenergic agents (clenbuterol, salmeterol) and a synthetic glucocorticoid (dexamethasone) were chosen to promote the activation of alternative pathways that could diminish TAG abnormal storage in NLSDM cells [35]. Indeed, beta-adrenergic agents stimulate activity of HSL, an enzyme that plays a central role in the activation of catecholamine-stimulated lipolysis [114] and that is able to hydrolyze TAG in vitro [115]. Conversely, dexamethasone significantly induces ATGL expression in adipocytes [112]. During metabolic pulse-chase experiments with 1-pyrenedecanoic acid, clenbuterol (1 µM), salmeterol (100 mM), and dexamethasone (1 µM) were added to the medium of NLSDM fibroblasts on day 1 and 6 of chase. While salmeterol and dexamethasone supplementation did not significantly decrease 1-pyrenedecanoic acid, clenbuterol treatment resulted in a marked diminution of this FA. As 1-pyrenedecanoic acid is incorporated into TAGs, its significant decrease suggested that clenbuterol could increase the rate of lipolysis. The difference between clenbuterol and salmeterol supplementation was probably due to the less selective and longer-acting effect of clenbuterol.

The metabolic deficiency in fibroblasts from NLSDM patients was corrected by overexpressing ATGL. The first patient had a homozygous deletion of four base pairs (c.799–802delGCCC), causing the production of a truncated protein (p.R268Pfs*50) [42]. The second subject was a homozygote for a missense mutation (p.R221P) that maintained residual ATGL activity [55]. The ATGL transfection (wild type) of NLSDM fibroblasts induced a marked decrease of cytoplasmic lipid storage, reverting the mutant cell phenotype [42,55]. Unfortunately, experiments testing therapeutic compounds on fibroblasts of NLSDM patients are very limited, and those on NLSDI are yet to be reported. Nevertheless, results obtained from the reported studies should stimulate the investigation of new drugs, taking into account that several lines of dermal NLSD fibroblasts are available on the Telethon Biobanknetwork for research purposes (www.biobanknetwork.telethon).

### 4.3. Induced Pluripotent Stem Cells

Induced pluripotent stem cells (iPSCs) are a unique cellular model that can be obtained directly from human adult somatic cells and that can be used to investigate the molecular bases of most disorders. In particular, iPSCs were generated as model for lysosomal storage diseases (e.g., Gaucher disease, mucopolysacharidosis 1H and IIIB) [116,117,118]. Disease-specific pluripotent human cells able to differentiate into the various affected tissues could provide new insights into disease pathophysiology [119,120,121,122]. Thus, they represent an opportunity to create a cellular model for those monogenic diseases for which there is a limited availability of relevant cell types [123]. NLSD is a group of pathologies reported in few subjects and, given the monogenic nature of these diseases, they are good candidate for iPSCs disease modeling [123].

**Figure 7:**
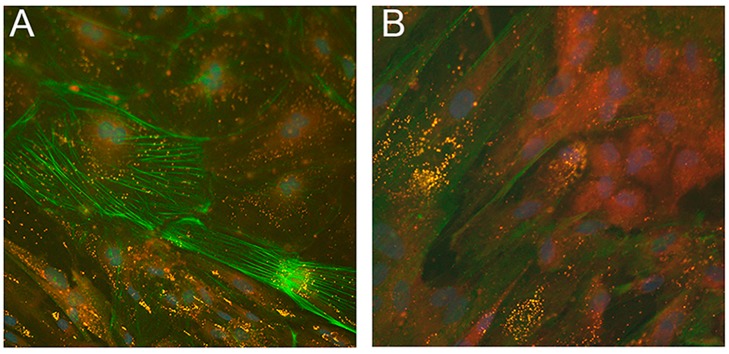
Neutral lipid storage in LDs of mesodermal cells from NLSDM embryoid bodies (EBs). (**A**,**B**) Immunostaining with primary anti-α-SMA (green) and with Nile Red (yellow) for neutral lipids showed a high number of LDs in EBs obtained from NLSDM iPSCs. Large LDs were present in many, but not all, mesodermal cells. Magnification: 40×.

Recently, two iPSC lines from NLSDM fibroblasts were obtained [124]. Molecular analysis showed that NLSDM iPSCs maintained *PNPLA2* mutations identified in the patient cells and real-time PCR studies revealed that they expressed pluripotent markers. These cells possess properties of embryonic-like stem cells and can differentiate into the three germ layers (ectoderm, mesoderm, and endoderm). Immunofluorescence evaluation of embryoid bodies (EBs), obtained from NLSDM iPSCs, also revealed abnormal lipid storage in the mesodermal cells (Figure 7). 

Finally, an evaluation of NLSDM pathophysiological features showed increased storage of TAG in NLSDM iPSCs compared to iPSCs generated from control cells. An oleic acid (OA) pulse-chase experiment confirmed that lipase activity was still impaired in NLSDM iPSCs.

These findings show that NLSDM iPSCs might represent autologous patient-specific stem cells which can be differentiated into (i) cardiomyocytes, in order to investigate the dysregulation of LD metabolism involved in the pathogenesis of cardiomyopathy; and (ii) myocytes and hepatocytes to investigate molecular mechanisms of muscle and hepatic damage.

## 5. Conclusions

Since the description of the first patients with an unusual accumulation of neutral lipids in different tissue types, fibroblasts obtained from these subjects were used to investigate the molecular defect underlying the metabolic disease. The beginning of the 21st century opened with the discovery of the gene sequences, *ABHD5* and *PNPLA2*, whose mutations cause the onset of the two neutral lipid storage diseases. The genetic identification represented a milestone for the diagnosis of these diseases, and, at the same time, a new impulse in the relatively young LD research field in which important basic questions are still not resolved. Biochemical investigations primarily focused on the ATGL lipase, its positive (ABHD5) and/or negative cofactors (G0S2, HIG2), the LD-associated proteins (PLINs), and on CIDEC and PEDF. However, molecular processes regulating lipid entry and release from LDs remain to be elucidated in many tissues. Furthermore, proteomic analyses identified a vast number of other proteins associated with LDs, but their functions are yet to be characterized. In NLSDM fibroblasts, preliminary results were obtained using some lipolytic compounds to overcome the ATGL defect, and these should also be tested in other tissue types, especially myocytes, cardiomyocytes, and hepatocytes that are affected by loss of function of ATGL or ABHD5.

Recent studies revealed that lipophagy releases FAs from LDs, a process where the interaction of ATGL and LC3 play pivotal roles; however, research on NLSDM cells is completely missing. Similarly, cells from NLSD patients represent a unique natural model to study lipid homeostasis and its alterations (lipotoxicity), a “non-energy storage role” of LDs, crucial for normal cell physiology. Future researches should exploit this biological resource to fill the gap in our knowledge.
